# The complete chloroplast sequence of *phalaenopsis malipoensis*, a rare orchidaceae species in China

**DOI:** 10.1080/23802359.2022.2107453

**Published:** 2022-08-17

**Authors:** Meijuan Hu, Enhui Tong, Yanping Zhang, Yinghui Cao, Yan Tong, Yangting Zhang, Wenjun Wang, Kai Zhao, Yuzhen Zhou

**Affiliations:** a College of Landscape Architecture, Fujian Agriculture and Forestry University, Fuzhou, China; b College of Life Sciences, Fujian Normal University, Fuzhou, China

**Keywords:** Chloroplast genome, Orchidaceae, *Phalaenopsis malipoensi*s, phyletic evolution

## Abstract

*Phalaenopsis malipoensis* Z.J. Liu et S.C. Chen, 2005 is a valuable species of Orchidaceae with significant ornamental value. This study reports the first complete chloroplast genome of *P.malipoensis* was collected integrally and clarified further. The genome was 144,528 bp in length and comprised four regions, including a pair of inverted repeats each 24,710 bp, a large single-copy region of 83,475 bp, and a small single-copy region of 11,633 bp. The whole genome contained 125 genes, including 8 rRNAs, and 39 tRNAs. Phylogenetic analysis indicated that *P. malipoensis* was closely related to *Phalaenopsis lobbii*. The analysis of *P. malipoensis* chloroplast genome will provide significant data for the identification and further improvement of *P. malipoensis*.

Orchidaceae is the largest family of monocotyledons plant family, with abundant floral resources, including five subfamilies (Chase et al. 2015). The genus *Phalaenopsis* has butterfly-like flowers, bright colors, and a long flowering period. It has the most popular and highly economical orchid species (Handini et al. 2016). It has a long history of cultivation, through years of breeding, has been cultivated with many good characters, and high ornamental values varieties (Antonetti et al. 2021). However, with the destruction of the native habitats and reckless human pilfering, the number of native populations has been constantly decreasing. The *Phalaenopsis malipoensis* Z.J. Liu et S.C. Chen, 2005 is a new species of *Phalaenopsis* found in Malipo County, Yunnan Province, China, 2005 (Liu et al. 2005). It has a three-lobed lip, both white and yellow, and is very similar to *Phalaenopsis lobbii* in flower shape and color. (Liu et al. 2005; Zhang et al. 2020). This precious and rare species was endemic to China, but the lack of detailed information and necessary conservation measures has hindered its exposure to the world. Therefore, we studied the entire chloroplast genome of *P. malipoensis* and mapped its genetic and genomic data for use in genetics, phylogeny, and breeding of the *Phalaenopsis*.

Samples of *P. malipoensis* for sequencing were collected from Yunnan Province, China, and the living plants were deposited at the National Orchid Conservation Center of Fujian Agriculture and Forestry University (26°20′21.3″N, 113°12′39.6″E, Voucher specimen: MLPHDL-YN2019-19A, Yuzhen Zhou, zhouyuzhencn@163.com). DNA was extracted from leaves by hexadecyltrimethylammonium bromide (CTAB) method. The clean reads were obtained by sequencing through the Illumina NovaSeq high-throughput sequencing platform. The sequencing generated about 16 GB of data, which was stored in the GenBank Sequence Read Archive (SRA) (accession number: SRR17270921). Low quality reads and adapters were removed using the Fastp v0.23.1 software with default parameters (Chen et al. 2018). Sequence assembly was performed using Get Organelle (core software SPAdes 3.1; assembly options: -R 15 -k 21, 45, 65, 85, 105) (Bankevich et al. 2012). The manual correction was done using Bandage v0.8.1 software, as previously reported (Wick et al. 2015). The refined genome was annotated by using the online facilities, CPGAVAS2 and Geneious Prime v2022.0.1 programming (reference: *Phalaenopsis lobbii*, NC_059699.1), followed by physical check for the corrections of explanation data. Complete chloroplast sequence of *P. malipoensis* (GenBank accession: OL623704) is 144,528 bp in length with an average GC content of 37%. It contains a large single-copy (LSC) region of 83,475 bp, a small single-copy (SSC) region of 11,633 bp and a pair of inverted repeat (IR) of 24,710 bp. It contains a total of 125 genes, including 8 rRNAs and 38 tRNAs.

To determine the location of the phylogenetic tree, the sequences of *P. malipoensis* were compared with other 21 chloroplast genomic sequences downloaded from the NCBI GenBank using MAFFT v7 (Katoh et al. 2019). Comparison of the *P. malipoensis* chloroplast genome to previously published data shows a high level of gene synteny with one publicly available genome of *P. Lobbii* (Zhang et al. 2020). Maximum likelihood tree was constructed with 1000 bootstrap replicates through the RAxML-HPC2 on XSEDE program available on the CIPRES online website (https://www.phylo.org/). The output files were visualized by EvolView v3 with *Zingiber officinale* (NC_044775.1) and *Curcuma longa* (NC_042886.1) as outgroups (Alexandros 2014; Balakrishnan et al. 2019). The result of the phylogenetic tree indicated that *P. malipoensis*, *P. Lobbii*, and *Phalaenopsis mannii* (NC_050940.1) were sister groups (Figure 1) (Chen et al. 2020). Thus, the chloroplast genome analysis of *P. malipoensis* provides supportive data to further research on conservation genetics, phylogeny, and molecular breeding of Orchids.

**Figure 1.**
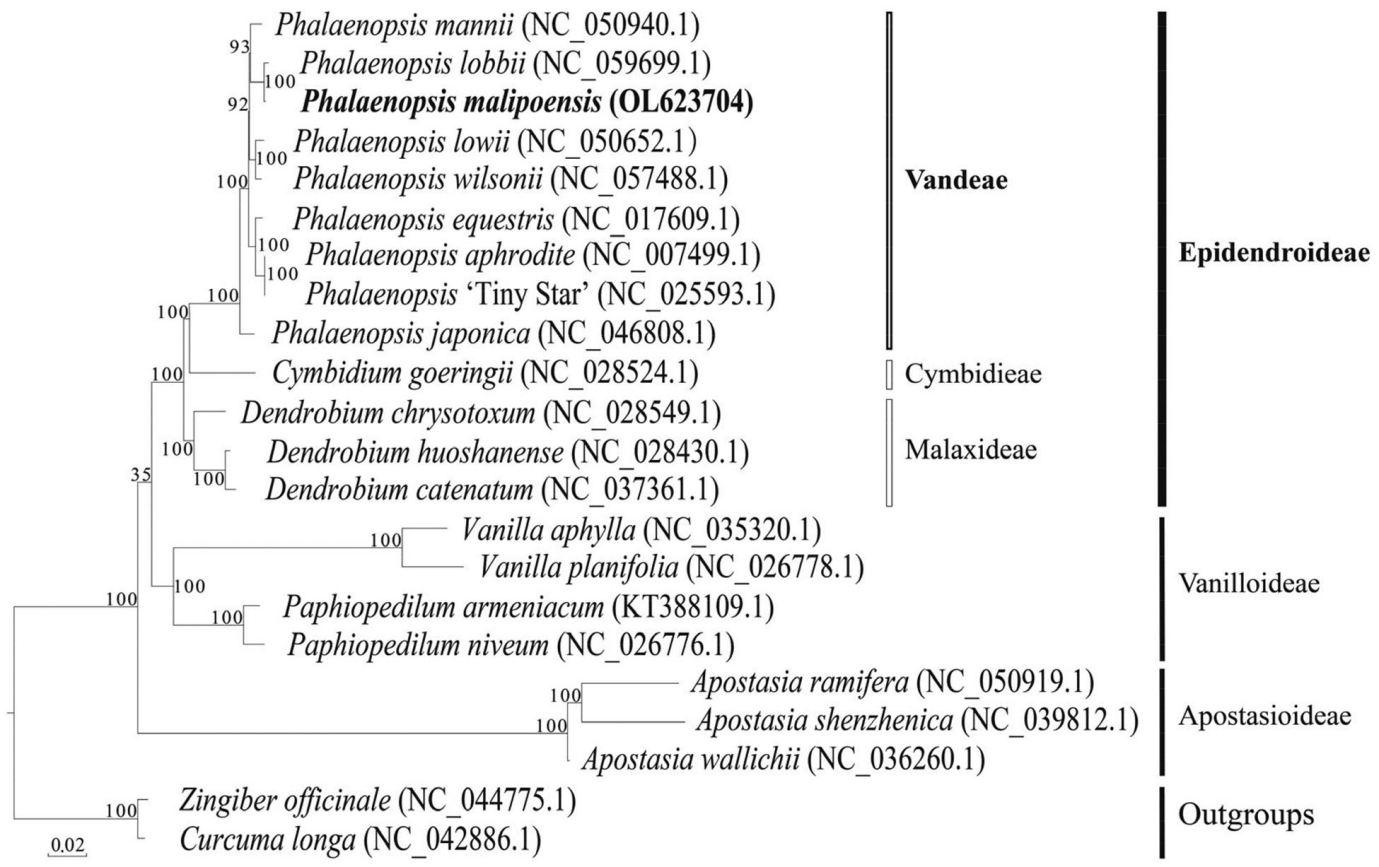
Maximum-likelihood phylogenetic tree include the complete chloroplasts of *Phalaenopsis malipoensis* (OL623704) and 19 other orchids with 2 outgroups, *Zingiber officinale* (NC_044775.1) and *Curcuma longa* (NC_042886.1). *P. malipoensis* is indicated in bold italic. The number on the branch node represents the bootstrap percentage of maximum parsimony, and the GeneBank accession number is shown after the scientific name of the species. Subfamilies of Orchidaceae are shown on the right.

## Data Availability

The genome sequence data that support the findings of this study are openly available in GenBank of NCBI at [https://www.ncbi.nlm.nih.gov] (https://www.ncbi.nlm.nih.gov/WebSub/) under the accession no. OL623704. The associated BioProject, SRA, and Bio-Sample numbers are PRJNA790458, SRR17270921, and SAMN24199872 respectively.

## Collection manager email

A specimen was deposited at the National Orchid Conservation Center of Fujian Agriculture and Forestry University (Yuzhen Zhou, zhouyuzhencn@163.com) under the voucher number MLPHDL-YN2019-19A.
